# Utilization of All-Chitin Composite Films for High-Density Three-Dimensional Cell Cultivation

**DOI:** 10.3390/molecules30214243

**Published:** 2025-10-31

**Authors:** Masayasu Totani, Mako Eda, Hiroyuki Shinchi, Jun-ichi Kadokawa

**Affiliations:** Graduate School of Science and Engineering, Kagoshima University, 1-21-40 Korimoto, Kagoshima 890-0065, Japan; m-totani@cb.kagoshima-u.ac.jp (M.T.); k5590749@kadai.jp (M.E.); hshinchi@eng.kagoshima-u.ac.jp (H.S.)

**Keywords:** all-chitin composite, cell morphology, cell network formation, three-dimensional cell cultivation

## Abstract

In this study, the potential of an all-chitin composite (AChC) film, prepared by dispersing and mixing high-crystalline scaled-down chitin nanofibers (SD-ChNFs) and low-crystalline chitin nanoparticles (ChNPs) in aqueous acetic acids, was evaluated for the formation of tissue-like cellular networks using a layered culture approach. The AChC film exhibited strong adhesion properties that enabled human cervical cancer (HeLa) cells to form a cellular network structure, whereas such adhesion and network formation were not observed with the individual components, SD-ChNFs and ChNPs. Furthermore, the cellular network structure was found to bridge the gaps between films, establishing three-dimensional (3D) connectivity. These findings demonstrate that AChC films serve as highly suitable functional biomaterials for constructing 3D tissue-like structures.

## 1. Introduction

Synthetic and natural polymers have been extensively investigated as scaffold materials for cell culture applications. Synthetic polymers are particularly valued for their ability to enhance mechanical strength and impart diverse biocompatible functions through precise control of molecular architecture [1,2]. However, their use requires careful consideration of the potential toxicity of degradation products and residual substances in vivo [3]. In contrast, natural polymers are widely employed in tissue engineering due to their biocompatibility and low toxicity [4,5,6]. For example, three-dimensional (3D) bioprinting has been developed to fabricate tissue-like constructs using natural polymers such as collagen, gelatin, and other extracellular matrix components [7,8,9]. Alternatively, natural polymer-based microscopic particles with adhered cells, known as cell beads, have been stacked to construct tissue-like structures [10]. This approach represents a “bottom-up” strategy for tissue construction, in contrast to conventional scaffold-based “top-down” methods [10,11]. Nevertheless, natural polymers such as collagen exhibit high hydrophilicity and therefore tend to form wettable structures like hydrogels. Moreover, their rapid degradation rates may limit their mechanical strength when used as scaffold materials [12,13].

Among natural polymers, the abundant structural polysaccharides chitin and cellulose have attracted significant attention in the biomedical field due to their ability to form nanomaterials, including nanofibers [14]. In particular, chitin-derived materials have been extensively studied for applications in regenerative medicine, such as skin wound healing and drug delivery, and some have already reached practical use and commercialization [15,16,17]. Chitin consists of linear chains of repeating *N*-acetyl-D-glucosamine (GlcNAc) units linked by *β*(1→4) glycosidic bonds. The acetamido groups at the C-2 position promote strong intermolecular hydrogen bonding, resulting in the high crystallinity and rigidity of chitin [18]. Although these properties confer structural stability, they also lead to poor solubility and limited processability, which have hindered the broader practical application of chitin.

To overcome these limitations, various strategies have been developed to fabricate chitin into nanofibrillar or nanoparticulate forms, thereby expanding its utility in biomaterials and tissue engineering [19,20,21]. Ionic liquids have been reported as effective solvents and dispersing media for chitin [22,23,24], offering the potential to develop new applications for this material. In previous work, it was found that the ionic liquid 1-allyl-3-methylimidazolium bromide serves as a dispersing medium for chitin to form ion gels. Furthermore, a simple bottom-up approach involving methanol addition was shown to regenerate chitin, enabling its self-assembly at the nanoscale into chitin nanofiber (ChNF) bundles hierarchically composed of thinner fibrils [24,25,26,27]. Treatment of the resulting self-assembled ChNF film with aqueous NaOH induced partial deacetylation of the GlcNAc units, yielding partially deacetylated (PDA)-ChNFs bearing amino groups [28,29,30,31]. The PDA-ChNFs were subsequently ultrasonically treated with aqueous acetic acid to dissociate the bundles through protonation and electrostatic repulsion, resulting in highly dispersible nanofibers with sizes ranging from 50 to 300 nm, referred to as “scaled-down ChNFs (SD-ChNFs)” with high crystallinity (crystalline index (CI), 90.5%) [27,31].

**Figure 1:**
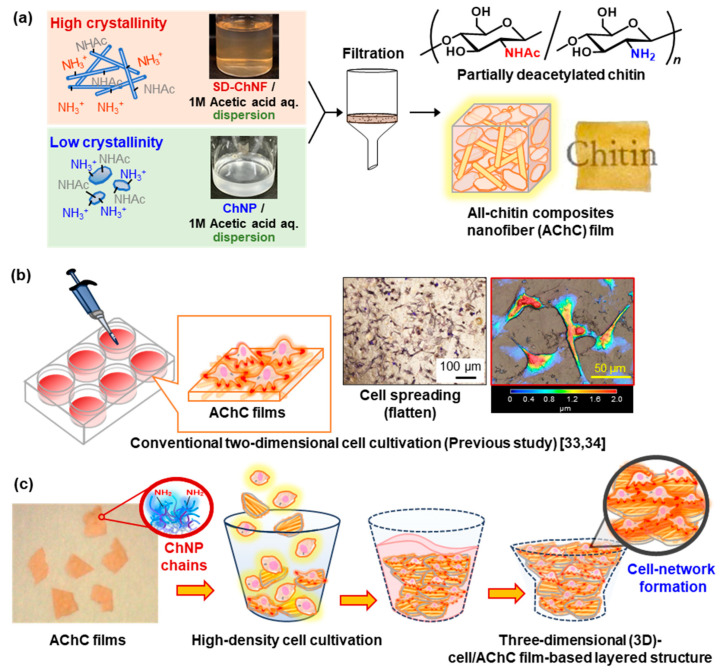
Schematic illustrations of (**a**) preparation of all-chitin composite (AChC) films [33,34], (**b**) conventional two-dimensional cell cultivation, and (**c**) three-dimensional (3D) cell/AChC film-based layered structure with high-density cell cultivation.

In contrast, low-crystalline chitin nanoparticles (ChNPs) were prepared through a two-step deacetylation of raw chitin powder using aqueous NaOH. In the first step (48 wt% aqueous NaOH, 4 °C), partial deacetylation and a reduction in crystallinity occurred [32], followed by a second step (30 wt% aqueous NaOH, 80 °C) that produced powdered ChNPs with a degree of deacetylation of 33% and a CI of 57.4% [33]. The resulting ChNPs exhibited good dispersibility in aqueous acetic acid. Consequently, the obtained ChNPs were used as a matrix, combined with high-crystalline SD-ChNFs, to fabricate an all-chitin composite (AChC) film (Figure 1a). The resulting AChC film is expected to serve as an eco-friendly material, providing a potential alternative to conventional plastics. Furthermore, a recent study revealed that immersion of the AChC film in water resulted in surface hydrophilization. This property enhanced cell adhesion, indicating the potential applicability of these films as biomaterials (Figure 1b) [34].

In conventional two-dimensional (2D) cell culture, cells typically exhibit an isolated, flattened, and elongated morphology on the substrate surface of the culture dish [35]. However, in native tissues, cells engage in extensive intercellular interactions and complex communication. The isolated morphology observed in conventional 2D cultures does not accurately recapitulate the cellular architectures present in vivo. This discrepancy raises concerns about the physiological relevance of such systems, particularly in the contexts of drug screening and tissue engineering. Consequently, there is an increasing demand for culture platforms that more faithfully replicate the native tissue microenvironment [35]. Various approaches have been explored to mimic biological tissues using chitin-based materials, including ChNFs. ChNFs have attracted considerable attention as scaffolds for cell culture because they can be assembled into fiber mat-like structures, providing a more defined 3D architecture. Moreover, precise control of fiber density and porosity is essential when using nanofibers as scaffolds. Although ChNF-based hydrogels have been widely explored as scaffolds, cells tend to distribute randomly on such structures, resulting in limited control over spatial organization and tissue-like formation. Furthermore, due to their inherently low mechanical strength, these hydrogels generally cannot maintain a self-supporting 3D structure.

One of the key challenges beyond previous studies is establishing effective cell–cell interactions, or cell–cell communication, within 3D scaffolds, which is the focus of the present investigation. When cells are cultured at high density on substrates with defined morphologies, particularly under subconfluent conditions, they have been shown to spontaneously organize into regular complexes interconnected by cellular networks [36,37]. This behavior is attributed to enhanced cell–cell communication facilitated by reduced intercellular distances. Consequently, in response to the underlying substrate, cellular morphology, proliferation, and collective behaviors are regulated, leading to a more organized, tissue-like state [38].

In this study, the potential of the AChC film to form 3D tissue-like structures was investigated based on the following main hypothesis: cells cultured at high density on AChC films may exhibit specific adhesion behaviors distinct from those observed in conventional 2D cultures, leading to the formation of tissue-like network structures. Consequently, self-organized cells bridging adjacent films are expected to give rise to 3D cell/AChC film-based layered structures (Figure 1c). Indeed, when AChC films were directly added to a cell suspension, the films with attached cells spontaneously accumulated in layers. After culture, adherent cells were fixed and stained to assess adhesion behavior, and the resulting cell/film-based layered structures were analyzed using laser microscopy (LM) and scanning electron microscopy (SEM).

## 2. Results

To fabricate the AChC film, SD-ChNF (CI = 90.5%) and ChNP (CI = 57.4%) dispersions in 1 M aqueous acetic acid were prepared following previously established procedures (supplementary materials) [31,33,34]. The two dispersions were then mixed at a weight ratio of SD-ChNFs to ChNPs of 1:6.6. This composition ratio has previously been shown to provide superior mechanical strength and cell adhesion properties [33,34]. The mixture was subjected to membrane filtration, followed by drying under reduced pressure, to yield the AChC film with a CI of 65.4% (supplementary materials). The SD-ChNF and AChC films were then cut into smaller, uniformly sized pieces for subsequent cultivation, as shown in Figure 1c. In contrast, the ChNPs with a CI of 60.5% did not form films and were therefore used in powder form.

**Figure 2:**
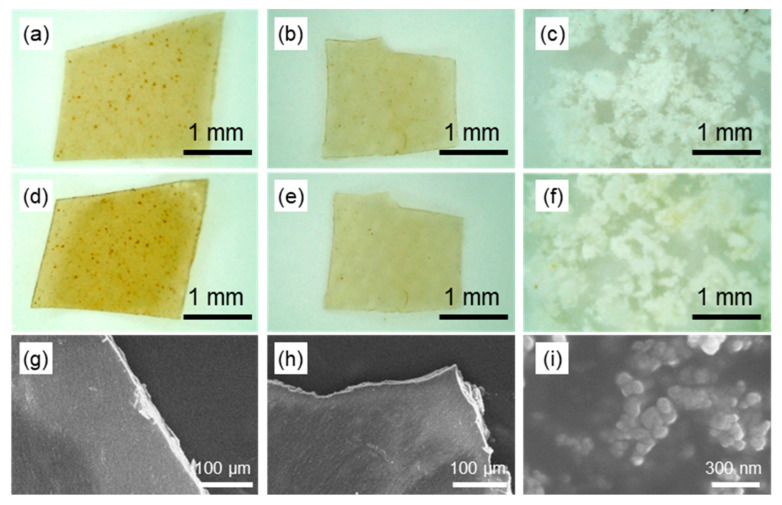
Photographs and SEM images of (**a**,**d**,**g**) SD-ChNF, (**b**,**e**,**h**) AChC, and (**c**,**f**,**i**) ChNP samples before and after autoclave sterilization: (**a**–**c**) before sterilization; panels and (**d**–**i**) after sterilization.

To evaluate stability in aqueous environments, SD-ChNF and AChC films, as well as ChNP powder, were immersed in sterilized water and subjected to autoclave sterilization (121 °C, 103 kPa, 20 min). Figure 2 presents photographs and SEM images; Figure 2a,d,g SD-ChNF, Figure 2b,e,h AChC, and Figure 2c,f,i ChNP samples before and after autoclave sterilization. The photographs in Figure 2a,b,d,e show that the SD-ChNF and AChC films maintained their integrity under high-temperature and high-pressure aqueous conditions without structural disruption. SEM observations in Figure 2g,h further confirmed that SD-ChNF and AChC films preserved flat surfaces without detectable microcrack formation. In contrast, Figure 2c,f,i show that the ChNPs remained as aggregated structures of approximately 50 nm in diameter, with no redispersion observed after autoclave sterilization. These results indicate that the materials exhibit remarkable structural stability in aqueous environments, sustained by strong hydrogen bonding among chitin chains, a distinctive thermal characteristic of chitin-based materials.

**Figure 3:**
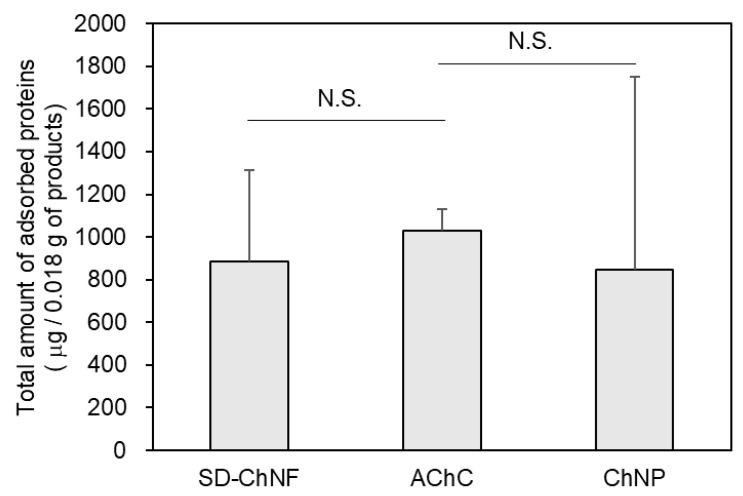
Total adsorption of FBS proteins on SD-ChNF and AChC films, and ChNP powder. Error bars represent standard deviations (n = 3). Statistical significance was determined by ANOVA, followed by Dunnett test; N.S. = Not significant.

Chitin is well known for its high biocompatibility, which facilitates favorable interactions with proteins [39,40]. To evaluate the interaction of serum proteins with each substrate, the adsorption of fetal bovine serum (FBS) proteins from cell culture medium was assessed. Figure 3 shows the total amounts of protein adsorbed on SD-ChNF and AChC films, as well as on ChNP powder. These results indicate that the protein-adsorbing properties of chitin are retained even after autoclaving. Moreover, protein adsorption appears to be minimally affected by the morphological differences among SD-ChNF, AChC films, and ChNP powder.

**Figure 4:**
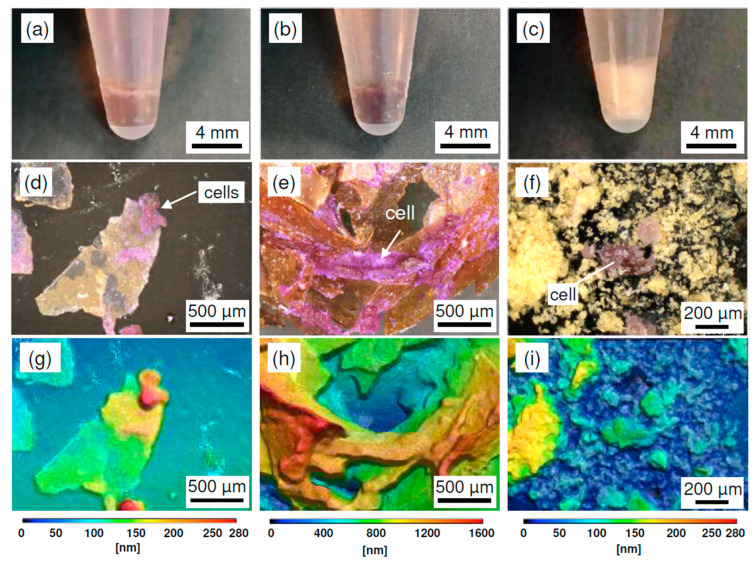
Photographic images, laser microscopy (LM) images, and three-dimensional (3D) height images obtained from LM of HeLa cell–substrate mixtures after 4 days of static cultivation on (**a**,**d**,**g**) SD-ChNF films, (**b**,**e**,**h**) AChC films, and (**c**,**f**,**i**) ChNP powder in E-MEM medium at 37 °C under 5% CO_2_.

To evaluate cell adhesion behaviors under high-density cultivation conditions, SD-ChNF and AChC films, as well as ChNP powder, were added to human cervical cancer (HeLa) cell suspensions. The cut SD-ChNF and AChC film pieces were approximately 10^4^ µm^2^ in size, whereas ChNPs consisted of particles much smaller than individual cells. From each 18 mg of substrate, SD-ChNF and AChC films supported cell densities of approximately 3 × 10^6^ cells cm^−2^, whereas ChNPs resulted in a cell density of only approximately 0.02 cells cm^−2^. SD-ChNF and AChC films, as well as ChNP powder, seeded with HeLa cells, were subjected to inversion mixing at 1-h intervals for the first 6 h. During this period, all substrates retained their dispersibility without aggregation or hydrogelation. The culture media were then removed, and the aggregated cell–substrate constructs were gently washed with phosphate-buffered saline (PBS). Figure 4 shows photographic images of HeLa cell/substrate mixtures after 4 days of static cultivation on (a) SD-ChNF films, (b) AChC films, and (c) ChNP powder, along with corresponding LM and 3D height images of fixed and crystal violet (CV)-stained adherent HeLa cells for (d,g) SD-ChNF films, (e,h) AChC films, and (f,i) ChNP powder. After 4 days of cultivation, HeLa cells had settled with SD-ChNF and AChC films, as well as ChNPs, at the bottom of the culture tubes. The specimens were then fixed with glutaraldehyde and stained with CV, removed from the tubes, and observed by LM. In Figure 4d, several cell aggregates adhered to the SD-ChNF films, as indicated by white arrows, and these aggregates retained their spherical shape (Figure 4g). In contrast, when cells were co-cultured with AChC films, numerous stained HeLa cells were distributed across multiple films (Figure 4e, white arrow). The adherent cells spread across the AChC films and lost their spherical morphology, as shown in Figure 4h. In the case of cells co-cultured with ChNP powder, isolated cell aggregates formed among the ChNP particles (Figure 4f,i).

**Figure 5:**
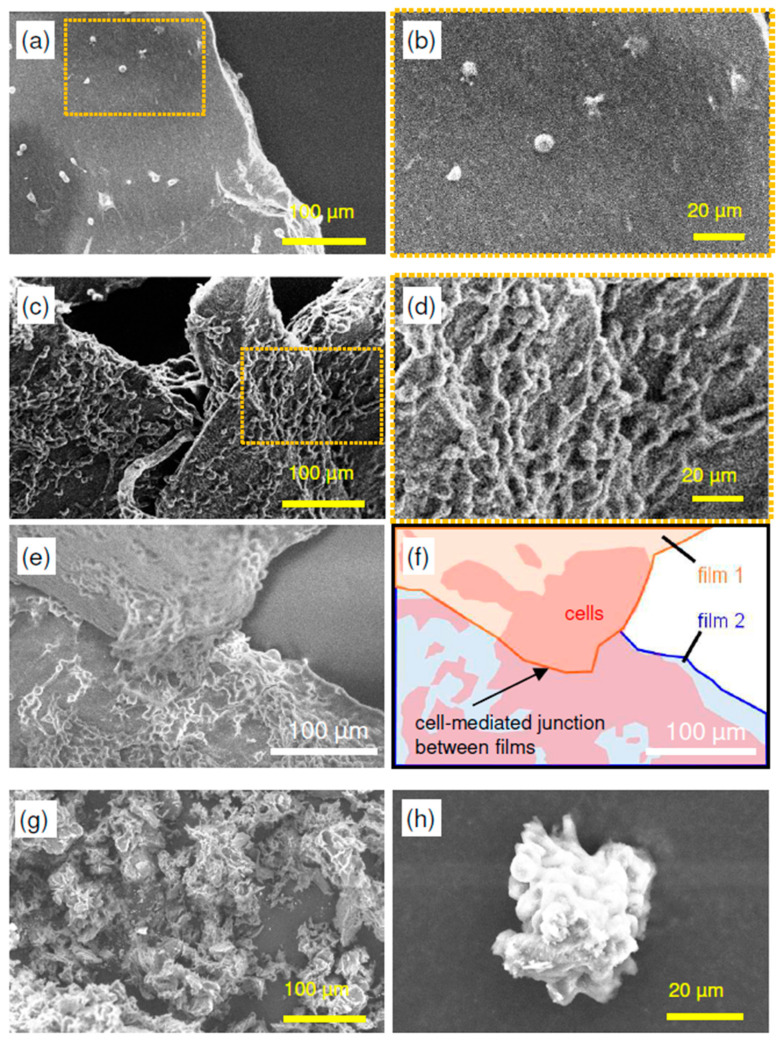
SEM images of adherent HeLa cells on (**a**,**b**) SD-ChNF films, (**c**–**e**) AChC films, and (**g**,**h**) ChNP powder. Panel (**f**) shows a schematic diagram corresponding to panel (**e**).

To investigate the morphology of HeLa cells adhered to each substrate in greater detail, SEM observations were performed. Figure 5 shows SEM images after cell cultivation: (Figure 5a,b) SD-ChNF films, (Figure 5c–e) AChC films, and (Figure 5g,h) ChNP powder. Figure 5f presents a schematic diagram corresponding to Figure 5e. HeLa cells adhered sparsely to the SD-ChNF films and did not exhibit elongated morphology (Figure 5a,b). In contrast, cells adhered extensively across the surfaces of AChC films, resulting in the formation of network-like structures (Figure 5c,d). To examine interactions between cells on adjacent AChC films in more detail, further observations were conducted. At the boundaries of the AChC films, cells formed physically interconnected networks, creating robust structures that linked the films (Figure 5e,f). In the case of the ChNP sample, HeLa cells adhered by wrapping around individual particles and forming small aggregates, but no dispersion across the sample was observed (Figure 5g). The cells were subsequently detached and analyzed by SEM to confirm the aggregated structures (Figure 5h). These results indicate that, although the molecular chains of ChNPs are believed to interact well with cells [34], strong adhesion between the powder and cells does not occur in the absence of a film-like surface. Network-like cellular structures form exclusively on AChC films because cells strongly adhere to the stiff film surface, promoting the development of intracellular cytoskeletal elements such as actin filaments, while densely packed neighboring cells generate mutual tension [36,37,41].

## 3. Discussion

The results of HeLa cell culture assays using AChC films under high cell density conditions demonstrated the formation of cellular network structures across multiple fragments of AChC films, indicating their potential for constructing 3D tissue-like structures. The remarkable cell adhesion observed on the AChC films is attributed to the optimal content of ChNP components, as reported in previous studies [34]. In contrast, when SD-ChNF films were used, cells either adhered locally as spheroids in suspension or only a few single cells attached sparsely. For ChNP powder, which cannot be processed into films, only small HeLa cell spheroids formed during culture. Therefore, both the SD-ChNF films and ChNP powder failed to function as scaffolds capable of supporting cell network formation.

**Figure 6:**
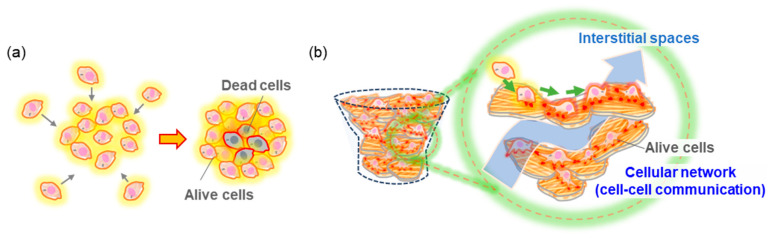
Schematic illustration of (**a**) a conventional cell spheroid and (**b**) the 3D cell/scaffold film-based structures developed in this study.

Considering the aforementioned results, 3D cell/scaffold film-based structures can be achieved through the cooperative assembly of cells and AChC films by stacking film fragments with high cell adhesion capability. We propose that 3D AChC film layered cultures can form 3D scaffolds with unique cell network structures that have not been observed using conventional methods. During cultivation on AChC films, cells adhere to the stiff film surface, elongate, and develop intracellular actin filaments, thereby establishing strong adhesion [36,37,41]. Furthermore, when cells are densely packed or overlap, cell–cell communication is promoted, facilitating the formation of network-like cellular architectures distinct from the adhesion behavior of isolated cells [37,38]. In previously reported 3D cell culture methods, spheroidal structures composed of densely packed cells were proposed as effective for short-term evaluations; however, their aggregated cellular structures raise concerns regarding potential cell death during long-term culture due to insufficient nutrient and oxygen supply (Figure 6a) [42].

In this study, AChC films were stacked with a design that maintains large interstitial spaces to allow adequate nutrient diffusion (Figure 6b). Consequently, the resulting morphology supports the formation of cell network structures even under high-density culture conditions. This 3D structural model is expected to support long-term culture and to exhibit distinct cellular characteristics compared with conventional scaffold materials, such as hydrogels and nanofiber mats.

## 4. Conclusions

In this study, SD-ChNF and AChC films, as well as ChNP powder, were evaluated for their potential in 3D film-based layered cell cultivation. LM observations confirmed that cells co-cultured with AChC films adhered extensively across multiple films. SEM analyses revealed that, under high cell density culture conditions, AChC films promoted the formation of cell networks not observed on SD-ChNF films or ChNP powder. These results indicate that AChC films enable the fabrication of 3D-cell/scaffold film-based layered structures. These findings are expected to advance the development of novel biomaterial surface designs and tissue engineering strategies through further in-depth studies using this approach. This culture strategy, which facilitates the establishment of 3D cellular networks, is anticipated to provide a physiologically relevant model that more closely mimics in vivo tissue architecture, offering significant utility for drug development and investigations into tumor tissue formation. Looking ahead, this study is anticipated to enable the construction of a miniature cancer tissue model, representing a first-of-its-kind system based on the current approach, with potential applications in drug development and developmental biology studies. Future efforts, alongside the pursuit of more complex tissue constructions, will require appropriate methods for observing internal structures and evaluating physiological activity.

## 5. Materials and Methods

### 5.1. Materials

AChC film was prepared according to a previously reported method (supplementary materials) [33]. HeLa cells (Accession Number: JCRB9004) were purchased from the Japanese Collection of Research Bioresources Cell Bank (JCRB Cell Bank, Osaka, Japan). Detailed information on the cells is available at: https://cellbank.nibn.go.jp/~cellbank/cgi-bin/search_res_det.cgi?ID=545 (accessed on 27 September 2025). Eagle’s Minimum Essential Medium (E-MEM), MEM essential amino acids solution (MEM-EA), penicillin-streptomycin solution (PS), and phosphate-buffered saline (PBS) were purchased from Fujifilm Wako. Fetal bovine serum (FBS) was obtained from Gibco, and trypsin-EDTA was purchased from Nakalai Tesque. A dual-range bicinchoninic acid protein assay kit (DR-BCA) was obtained from Energenesis Biomedical Co., Ltd., (Taipei, Taiwan). For the experiments, SD-ChNF and AChC films were cut into small pieces, and ChNP powder was placed into sterile 1.5 mL microcentrifuge tubes with water. All samples were sterilized by autoclaving (NCC-1701, As-one, Osaka, Japan).

### 5.2. Protein Adsorption Test

The sterilized samples (18 mg) were immersed in 0.5 mL of cell culture medium containing 10% FBS. After incubation at 37 °C for 24 h, the samples were gently washed with PBS. To remove adsorbed serum proteins from the sample surfaces, the films were immersed in 1 mL of 0.1 wt% sodium dodecyl sulfate (SDS) solution and sonicated for 30 min. The concentration of serum proteins in the SDS solution was determined using the test-tube procedure of a dual-range-BCA assay. Absorbance was measured at approximately 562 nm, and a standard curve was prepared using known concentrations of bovine serum albumin.

### 5.3. Cell Adhesion Test

Cells were pre-cultured in two 75 cm^2^ culture flasks until reaching a subconfluent state. They were then washed once with PBS and treated with a trypsin-EDTA solution. After centrifugation at 700 rpm (300× *g*) for 5 min, the cells were resuspended in E-MEM supplemented with 10% FBS, 1% E-MEM EA, and 1% PS. Subsequently, cell culture media (3 × 10^6^ cells mL^–1^) were prepared in sample tubes containing 1.0 mL of E-MEM supplemented with 10% FBS and 1% E-MEM EA. Sterilized samples (18 mg) were added to the cell suspensions and mixed by inversion at 1-h intervals for the first 6 h at 37 °C under 5% CO_2_. The mixtures were then incubated for 4 days at 37 °C under 5% CO_2_. After incubation, the samples were gently washed with PBS to remove non-adherent cells. Adherent cells were fixed with 2 wt% glutaraldehyde in PBS for 1 day at room temperature, followed by staining with 0.01 wt% crystal violet in methanol for 1 day. After staining, the samples were washed thoroughly with deionized water. Adherent cells were observed using LM and SEM.

### 5.4. Measurements

The CIs of the chitin crystals were calculated using a method reported in the literature [43], based on powder X-ray diffraction data. UV-vis spectroscopy for the protein adsorption assay was performed using a V-550 spectrophotometer (JASCO Co., Tokyo, Japan). Adherent HeLa cells were observed with a VK-X3000 LM (Keyence Co., Osaka, Japan) and a TM4000Plus II tabletop SEM (Hitachi High-Tech Co., Tokyo, Japan).

## Data Availability

The original contributions presented in this study are included in the article. Further inquiries can be directed to the corresponding author.

## Supplementary Materials

The following supporting information can be downloaded at: https://www.mdpi.com/article/10.3390/molecules30214243/s1, Preparation of SD-ChNF dispersion and film; Preparation of ChNP powder; Preparation of AChC film.
