# Supplementary material for: Utilization of All-Chitin Composite Films for High-Density Three-Dimensional Cell Cultivation
Source: Molecules. 2025 Oct 31;30(21):4243. doi: 10.3390/molecules30214243 (PMC12608466; doi:10.3390/molecules30214243)
Supplement: Supplementary file 1 [file molecules-30-04243-s001.zip › molecules-3923089-supplementary.pdf]

Supplementary Materials

Utilization of All-Chitin Composite Films for High-Density Three-Dimensional Cell Cultivation

Masayasu Totani[1], Mako Eda[1], Hiroyuki Shinchi[1] and Jun-ichi Kadokawa[1]*

[1]Graduate School of Science and Engineering, Kagoshima University, 1-21-40 Korimoto, Kagoshima 890-0065, Japan

The high-crystalline scaled-down chitin nanofibers (SD-ChNFs), low-crystalline chitin nanoparticles (ChNPs), and all-chitin composite (AChC) films were prepared according to previously reported methods. The results of their NMR and XRD measurements are available in the previously published papers [1-3].

1.1. Preparation of SD-ChNF dispersion and film [1,2]

A mixture of chitin (0.120 g, 0.59 mmol) with AMIMBr (1.00 g, 4.92 mmol) was allowed to stand for 24 h at room temperature and then heated with stirring for 24 h at 100 °C to obtain a chitin ion gel (10 wt%). This gel was then immersed in methanol (30 mL) for 72 h at room temperature for regeneration, followed by ultrasonication (Branson 1510 (42 kHz, 70 W)) for 10 min to produce a self-assembled ChNF dispersion with methanol. The resulting dispersion was

subjected to suction filtration to separate the ChNFs, which were washed with methanol and dried under reduced pressure to obtain a self-assembled ChNF film. After a mixture of the resulting ChNF film (0.120 g, 0.59 mmol) with aqueous NaOH (30 wt%, 20 mL) was heated for 5 h at 80 °C, the deacetylated material was isolated by suction filtration, immersed in water (30 mL) for 10 min via ultrasonication (Branson 1510 (42 kHz, 70 W)), filtered, washed with water, and dried under reduced pressure to obtain a PDA-ChNF film. A mixture of the PDA-ChNF film (80.0 mg, 0.41 mmol) with aqueous acetic acid (1 M, 20 mL) was ultrasonicated using a homogenizer (Branson Advanced-Digital Sonifier 450; 20 kHz, 400 W) for 10 min at room temperature to produce a SD-ChNF dispersion. For preparing a SD-ChNF film, the abovementioned dispersion was subjected to suction filtration and the residue was subsequently washed with water and dried under reduced pressure.

1.2. Preparation of ChNP powder [2,3]

A mixture of chitin (0.100 g, 0.49 mmol)with aqueous NaOH (48 wt%, 20 mL)was incubated for 24 h at 4 °C according to method adapted from the literature [4]. After neutralization with 1 M hydrochloric acid, the dispersed product was isolated by suction filtration, immersed in excess

water, and filtered to be a partially deacetylated low-crystalline chitin in a hydrated gel state. The resulting gelling material was lyophilized to yield PDA-LC-Ch film (degree of deacetylation = 24% for the total repeating units, by ^{1}H NMR spectrum). The obtained PDA-LC-Ch (98.7 mg) was further treated with aqueous NaOH (30 wt%, 20 mL) for 5 h at 80°C with stirring and subsequently dispersed with ultrasonication using a homogenizer (Branson Advanced-Digital Sonifier 450; 20 kHz, 400 W). The resulting dispersion was then filtered, and the filtrate was dried under reduced pressure to obtain ChNP powder (95.0 mg).

1.3. Preparation of AChC film [2,3]

A typical experimental procedure for AChC was as follows (SD-ChNF/ChNP weight ratio = 1/6.6). A mixture of the PDA-ChNF film (5.3 mg) with 1 M aqueous acetic acid (10 mL) was ultrasonicated using a homogenizer (Branson Advanced-Digital Sonifier 450; 20 kHz, 400 W) for 10 min at room temperature to produce a SD-ChNF dispersion. Separately, a mixture of the ChNP powder (14.8 mg) with 1 M aqueous acetic acid (5.0 mL) was ultrasonicated using a homogenizer (Branson Advanced-Digital Sonifier 450; 20 kHz, 400 W) for 20 min at room temperature to produce a ChNP dispersion. The SD-ChNF and ChNP dispersions were then mixed with stirring.

After suction filtration of the resulting mixture was conducted, the residue was washed with water and dried for 2 h at 60 °C under reduced pressure to produce AChC film.

Reference:

[1] Egi, Y.; Kontani, A.; Kadokawa, J. Fabrication of all-chitin composite films, *Int. J. Biol. Macromol.* **2023**, *253*, 127512, https://doi.org/10.1016/j.ijbiomac.2023.127512.

[2] Totani, M.; Tanihata, Y.; Egi, Y.; Kadokawa, J. Fabrication of self-reinforced chitin composites by double crystalline blend approach. *Int. J. Biol. Macromol.* **2025**, *286*, 138441. https://doi.org/10.1016/j.ijbiomac.2024.138441.

[3] Totani, M.; Shinchi, H.; Kadokawa, J. Cancer cell adhesion property on all-chitin composite films with reduced crystallinity. *Carbohydr. Res.* **2025**, *549*, 109373. https://doi.org/10.1016/j.carres.2024.109373.

[4] Sato, K.; Noguchi, T.; Kiyose, M. Deacetylation and gelation of amorphous chitin cake. *Chitin Chitosan Res.* **2019**, *25*, 136–137.
